# Immunohistochemistry in postmortem diagnosis of acute cerebral hypoxia and ischemia: A systematic review

**DOI:** 10.1097/MD.0000000000026486

**Published:** 2021-06-25

**Authors:** Rosario Barranco, Alessandro Bonsignore, Francesco Ventura

**Affiliations:** Department of Legal and Forensic Medicine, University of Genova, Via De Toni 12, Genova, Italy.

**Keywords:** acute cerebral hypoxia, acute cerebral ischemia, autopsy, forensic pathology, immunohistochemistry

## Abstract

**Background:**

: Discovery of evidence of acute brain ischemia or hypoxia and its differentiation from agonal hypoxia represents a task of interest but extremely difficult in forensic neuropathology. Generally, more than 50% of forensic autopsies indicate evidence of brain induced functional arrest of the organ system, which can be the result of a hypoxic/ischemic brain event. Even if the brain is the target organ of hypoxic/ischemic damage, at present, there are no specific neuropathological (macroscopic and histological) findings of hypoxic damage (such as in drowning, hanging, intoxication with carbon monoxide) or acute ischemia. In fact, the first histological signs appear after at least 4 to 6 hours. Numerous authors have pointed out how an immunohistochemical analysis could help diagnose acute cerebral hypoxia/ischemia.

Data sources: This review was based on articles published in PubMed and Scopus databases in the past 25 years, with the following keywords “immunohistochemical markers,” “acute cerebral ischemia,” “ischemic or hypoxic brain damage,” and “acute cerebral hypoxia”.

**Objectives:**

: Original articles and reviews on this topic were selected. The purpose of this review is to analyze and summarize the markers studied so far and to consider the limits of immunohistochemistry that exist to date in this specific field of forensic pathology.

**Results:**

: We identified 13 markers that had been examined (in previous studies) for this purpose. In our opinion, it is difficult to identify reliable and confirmed biomarkers from multiple studies in order to support a postmortem diagnosis of acute cerebral hypoxia/ischemia. Microtubule-associated protein 2 (MAP2) is the most researched marker in the literature and the results obtained have proven to be quite useful.

**Conclusion:**

Immunohistochemistry has provided interesting and promising results, but further studies are needed in order to confirm and apply them in standard forensic practice.

## 1 Introduction

Discovery of evidence of acute brain ischemia or hypoxia and its differentiation from agonal hypoxia represents a task of particular interest but extremely difficult in forensic neuropathology.^[1–2]^

Generally, more than 50% of forensic autopsies indicate evidence of brain induced functional arrest of the organ system, which can be the result of a hypoxic/ischemic brain event.^[3]^ The latter can be caused by multiple conditions such as traumatic or chemical events, respiratory and cardiac arrest, asphyxiation or obstruction of the cerebral or cervical vessels.^[3]^

At present, there are still no specific neurological signs in case of acute fatal hypoxia (which may occur, for example, in strangulation or drowning).^[1,2,4]^

Ischemia and hypoxia have often been considered of a similar nature and define the cell's inability to receive and use oxygen.^[5,6]^ However, ischemia is characterized by the reduction/absence of cerebral blood flow resulting in irreversible neuronal destruction while hypoxia foresees a lack of oxygen in the blood and indicates an increase in cerebral blood flow with a reversible alteration of brain functions.^[5,7]^ Specifically, in the event of the obstruction of the areas (aspiration, asthma, etc.) or absence of ambient oxygen (as in drowning), cerebral circulation continues but an increase in plasma carbon dioxide will occur (with a secondary dilation of the arteries) as well as a reduction in the partial pressure of oxygen (pO_2_).^[5,6]^ The obstruction of the cerebral vessels or of the neck (as in the case of hanging or strangulation) induces both a reduction/arrest of the intracranial circulation as well as an alteration of the values of gases in the blood and the pH associated with acidosis and increase in lactates.

As a result of these processes, a dysfunction of the sodium-potassium pump and cytotoxic edema occurs.^[5]^ In the case of reperfusion, the cytotoxic edema will be associated with vasogenic edema within 10 to 20 minutes.^[5]^

When analyzing ischemic-hypoxic brain damage, the susceptibility of specific brain areas (watershed area) must be considered in relation to vascular anatomy and in relation to the different types of neurons.^[8,9]^ In this sense, the fissure between the first and second turns of the frontal lobe, the CA1 region of the hippocampus, the Purkinje cells of the cerebellum and the pale globe represent the areas that are most susceptible to hypoxia and samples should be taken.^[5,8]^

Some neurobiological changes occur in neurons after hypoxic/ischemic damage but histological neuronal findings appear in the brain after several hours. On the other hand, neuronal necrosis and neuron loss are detectable after a long period of survival.^[10–15]^

If death occurs within a few hours, it is extremely complicated (if not impossible) to observe specific macroscopic changes.^[16,17]^ Even from a microscopic point of view, the very acute ischemic/hypoxic area of the brain is difficult to trace.^[18]^ Ischemic neurons initially reveal collapsed, shrunken and pyknotic nuclei and intensely eosinophilic cytoplasm (in hematoxylin-eosin staining),^[5,19]^ and the Nissl substance appears dispersed and finely granular. Subsequently, when chromatin has degraded, the nuclei become more eosinophilic and appear to merge with the surrounding cytoplasm. Neutrophils can be mildly vacuolized (nonspecific relief) or normal.^[19]^ These changes (the first to occur in case of necrosis) are histologically visible only after a survival time of at least 4 to 6 hours or even 8 to 12 hours.^[16,18–20]^ In many cases, however, death occurs within hours or even minutes of the initial acute event.

If death occurs after several minutes (as in cases of drowning or hypoxia) or within just a few hours, it would not be possible to identify ischemic/hypoxic brain damage through a conventional macroscopic and histological examination.

After the initial ischemic/hypoxic damage, a series of structural alterations of proteins of the brain begins to take place. In this regard, several immunohistochemical markers have been investigated with the objective of identifying acute brain damage. Currently, a routine method that could resolve this problem has not been identified.

On the basis of a critical analysis of the literature on the use of immunohistochemistry in hypoxic-ischemic injury, this review aims to analyze and summarize all the principal markers examined to date, regarding acute ischemic/hypoxic brain damage.

## 2 Materials and methods

All the main scientific studies regarding the immunohistochemical evaluation of acute cerebral hypoxia/ischemia were examined. Specifically, we used the search engines PUMED (https://www.ncbi.nlm.nih.gov/pmc/) and Scopus (https://www.scopus.com) to research the keywords “immunohistochemical markers,” “acute cerebral ischemia,” “ischemic or hypoxic brain damage,” and “acute cerebral hypoxia”. All major papers published in the English language in the last 25 years were also considered. The results of the search were screened on the basis of the titles and abstracts of the papers. We excluded papers that did not fully relate with the subject under examination: those that did not relate with the immunohistochemical diagnosis of acute hypoxic/ischemic brain injury. Only studies that considered an acute ischemic/hypoxic brain damage were considered and included in the review. Studies where the period of survival was greater than 12 to 24 hours were not taken into consideration. Articles deemed relevant to the issue under investigation were read and analyzed in their entirety. Furthermore, studies with mainly forensic purposes were mainly examined. We conducted a critical analysis of all the scientific papers selected, examining all the markers utilized and the immunohistochemical response.

## 3 Results

The search yielded over 35 scientific papers deemed suitable for analysis. We identified the following markers that had been examined (in previous studies) for the purpose of diagnosing acute cerebral hypoxia and ischemia (Table 1). The principal markers were:

**Table 1:** Immunohistochemistry in the postmortem diagnosis of acute cerebral hypoxia and ischemia: results for the evaluated immunohistochemical markers.

Marker	Analyzes	Consideration
Tau protein	Salama et al^[21]^ studied Tau expression in the animal models of acute hypoxia-ischemia.	Tau aggregates were significantly greater in hypoxic-ischemic models than in controls. The study has several limitations: the limited number of cases studied, a long period of survival.
S-100	Li et al^[24]^ studied this protein in the cerebral cortex in forensic autopsy cases.	The expression was lower in acute deaths from strangulation/hanging and drowning than in other groups
Calbindin-D28K	Bartschat et al^[2]^ analyzed the cerebellar expression of calbindin-D28k (CaBP-D28k) in forensic autopsy cases of acute hypoxia such as drowning or asphyxia.	Significant reduction in the expression of CaBP-D28k in cases of acute cerebral hypoxia compared to the control groups.
HIF-1	The cerebellar expression of HIF-1 alpha was evaluated in forensic autopsy cases of acute hypoxia such as drowning or asphyxia.^[2]^	HIF-1a staining revealed weak positive immunostaining in all cases (including in control cases).
VEGF	Expression of VEGF was analyzed in forensic autopsy cases of acute hypoxia such as drowning.^[2]^	The comparison between the groups did not indicate any change in immunoreactivity in cerebellar PCs due to hypoxic events.
Cox-2	Sanz et al^[50]^ studied the brain expression of Cox-2 in mice 6, 12, and 24 h after the occlusion of the middle cerebral artery.	This marker could be useful. However, further confirmatory studies are needed.
C-fos	The expression of c-fos in mice was analyzed after 6, 12, and 24 h from occlusion of the middle cerebral artery ^[50]^	The expression of c-fos increased in and around the ischemic lesions
HSP70	Kitamura^[10]^ analyzed the expression of HSP70 in autopsy cases of hypoxic/ischemic brain damage. Sanz et al^[50]^ researched the expression of HSP70 in mice after MCA occlusion. A further neuropathological study on forensic cases^[60]^ was proposed	The immunohistochemical expression was found the event of long-term survival.
MAP2	Several Authors analyzed the immunohistochemical expression of MAP2 in a forensic autopsy case of hypoxia/ischemia.^[1,62–66]^	MAP2 is considered a very early marker of ischemic neuronal damage, as demonstrated by the loss of neuronal MAP2 immunoreactivity from the results of experimental studies.^[1,62–66]^
SMI 32	Leifer et al^[54]^ conducted an immunohistochemical study on autopsy cases of cerebral hypoxia/ischemia.	The expression of SMI32 was predominantly reduced in cases of acute ischemia
Albumin	Løberg et al^[69]^ conducted a study of experimental animal samples (together with several autopsy cases) in order to understand if an uptake of plasma proteins occurs in damaged neurons after ischemic/hypoxic damage. Albumin expression after ischemic/hypoxic damage has been studied by Maeda et al^[70]^ in experimental animal samples	The application in the Forensic setting appears limited and not considered very useful.
GFAP and Vimentin	These markers have been analyzed and discussed in several previous studies^[1,10,54,73]^	GFAP and vimentin play a vital role if the survival time is prolonged for, they are linked to the reaction of glial cells to hypoxic/ischemic damage. The application in the Forensic setting appears limited and not considered very useful. Further confirmatory studies are needed.

Tau protein: Tau protein plays an important role in the assembly and stabilization of microtubules. In addition, this protein is involved in the mechanisms of signal transduction, interaction with the cytoskeleton actin, neuritis outgrowth, and stabilization during brain development.^[21,22]^ An alteration of the Tau protein has been found in neurodegenerative diseases.^[21]^ Other studies^[21,23]^ correlate the aggregates of this protein with acute brain damage such as ischemia. Salama et al^[21]^ studied Tau expression in the animal models of acute hypoxia-ischemia. Tau aggregates were significantly greater in hypoxic-ischemic models than in controls. According to the authors, this protein could be useful in the forensic investigation of asphyxiated death. However, the study has several limitations: the limited number of cases studied, a long period of survival (1 day), the unlikelihood of confirming whether this result is a direct effect of hypoxia or an epiphenomenon linked to secondary injury cascade.

S-100: S-100 protein is a binding-calcium protein with a subunit of A and B.^[24–28]^ The S100B subunit is highly specific for ependymocytes, oligodendrocytes and astrocytes in the central nervous system. On the other hand, the S100A subunit is present in skeletal muscles, lungs, liver, kidneys, pancreas, and heart.^[24–31]^ S100B has been clinically studied as a serum marker of brain damage.^[24,32–40]^ Li et al^[24]^ studied the immunohistochemical expression of the S100 protein in the cerebral cortex in forensic autopsy cases. A lower expression was seen in cases of asphyxia due to neck compression (strangulation and hanging) and drowning (than in other groups). The decrease in the number of S100-positive astrocytes was more evident in the cerebral cortex. No significant change in oligodendrocyte positivity was revealed. According to the authors, these results are suggestive of astrocytic diffuse damage (especially in the cerebral cortex) due to cerebral hypoxia and/or ischemia.^[24]^

Calbindin-D28K (CaBP-D28k): this protein is part of the EF-hand calcium-binding protein family and is expressed in the cytoplasm of neurons in many brain regions, including the Purkinje-cells of the cerebellum (PCs).^[2,41]^ Bartschat et al^[2]^ analyzed the cerebellar expression of CaBP-D28k in forensic autopsy cases of acute hypoxia such as drowning or asphyxia. In this study, the immunohistochemical analysis revealed a significant reduction in the expression of CaBP-D28k in cases of acute cerebral hypoxia compared to the control groups (polytrauma, heart failure). According to the authors, the discovery of a reduction in the concentration of calbindin-D28k could support the diagnosis of acute hypoxia.

HIF-1 alpha: Hypoxia-inducible factor controls the expression of the genes involved in the hypoxic response.^[28,42–44]^ HIF-1 alpha is almost absent in normoxia and promotes the expression of vascular endothelial growth factor (VEGF) in order to maintain homeostasis in hypoxic conditions.^[2]^ In 1 study^[2]^ the cerebellar expression of HIF-1 alpha was evaluated in forensic autopsy cases of acute hypoxia such as drowning or asphyxia. As a control group, cases of polytrauma and heart failure were selected. HIF-1a staining revealed weak positive immunostaining in all cases (including in control cases). Therefore, according to the authors, this marker is not deemed useful in the diagnosis of cerebral hypoxia.

VEGF: the protein plays an important role in angiogenesis and vascular permeability.^[45–47]^ VEGF expression is promoted by HIF, stimulating neovascularization in the hypoxic/ischemic brain area.^[2,48–49]^ Authors^[2]^ analyzed the cerebellar expression of VEGF in forensic autopsy cases of acute hypoxia such as drowning. Cases of polytrauma and heart failure were selected as a control group. According to the authors, the immunoreaction of VEGF was consistently negative. The comparison between the groups did not indicate any change in immunoreactivity in cerebellar PCs due to hypoxic events. The study proposed by Bartschat et al^[2]^ did not demonstrate that VEGF is induced during early responses to brain ischemia/hypoxia.

Cyclooxygenase-2 (Cox-2): it is an enzyme involved in the metabolization of arachidonic acid into prostanoids.^[50,51]^ The expression of Cox-2 is present in several brain areas (especially in the hippocampus and cerebral cortex) in normal conditions [5,19d3] and can be considerably induced under certain stimuli such as ischemia.^[50,52]^ In an experimental study, Sanz et al^[50]^ studied the brain expression of Cox-2 in mice 6, 12, and 24 hours after the occlusion of the middle cerebral artery (MCA). According to this study, an expression of Cox-2 was detected 6 to 24 hours from ischemic insult. At 6 hours this marker was identified within the MCA territory. At 24 hours the expression was restricted to the perifocal cortical area which indicated a high level of immunoreactivity. At 6 hours, Cox-2 was identified mainly in layer II of the ipsilateral cortex and rarely in striated neurons. At 24 hours, the immunoreactivity of Cox-2 was detected in the ipsilateral peripheral areas (layer II in the cingulate frontal cortex) surrounding the ischemic area.

C-fos: it is a proto-oncogene involved in numerous cellular functions. The transient activation of c-fos follows a cortical brain injury of various nature.^[53]^ In a previous study^[50]^ the expression of c-fos in mice was analyzed after 6, 12, and 24 hours from occlusion of the middle cerebral artery (MCA). According to this study, the immunoreactivity of this marker was observed after 6 hours primarily in the superficial layers of the cortex within the MCA territory. Within 24 hours, the expression of c-fos was seldom identified within the MCA territory but was very distinct in the ipsilateral undamaged cortex, primarily in the superficial layers of the cingulate frontal cortex. According to a further study,^[54]^ the expression of c-fos had increased in and around the ischemic lesions (autopsy case studies were limited to only 6 cases).

Shock Heat shock protein 70 (HSP70): it plays a central role in cellular repair and adaptation to stress.^[55–58]^ HSP70 protects cells from a host of stresses, including heat, hypoxia and oxidative stress.^[55,59]^ Kitamura^[10]^ analyzed the expression of HSP70 in autopsy cases of hypoxic/ischemic brain damage. According to this study, the immunohistochemical expression of HSP70 was found in the hippocampal regions CA2, CA3, and CA4 principally in the event of long-term survival after severe toxic or ischemic injury. In another study, Sanz et al^[50]^ researched the expression of HSP70 in mice after MCA occlusion. According to this study, 6 hours after the occurrence of ischemia, an immunoreactivity of HSP70 was observed after 6 hours (survival time). The intensity of the Hsp70 expression increased from 6 to 24 hours after the ischemic insult. Also at 24 hours, a strong expression of Hsp70 was identified in the neurons surrounding the ischemic area (penumbra like-zone). A further neuropathological study on forensic cases^[60]^ revealed an expression of HSP70 in areas CA2, CA3, and CA4 primarily in long survival time after severe hypoxic/ischemic damage.

Microtubule-associated protein 2 (MAP2): microtubule-associated proteins are the largest group of cytoskeleton proteins and play an important role in neuronal morphogenesis.^[1]^ MAP2 is the most abundant MAP family protein in the brain. This protein increases the elongation of the microtubules and reduces the rapid shortening frequency even if simply modifying but not prohibiting their dynamic behavior.^[1,61]^ MAP2 is considered a very early marker of ischemic neuronal damage, as demonstrated by the loss of neuronal MAP2 immunoreactivity from the results of experimental studies.^[1,62–66]^ In essence, studies in rats and gerbils have revealed results of an early loss of MAP2 immunoreactivity following ischemia, but there have been a limited number of studies on the human brain,^[1,54,67]^ and none of these studies refer to a broader number of cases comprised of different causes of ischemic/hypoxic brain death. Kuhn et al^[1]^ studied the immunohistochemical expression of MAP2 in a forensic autopsy case of hypoxia/ischemia (including cases of drowning and hanging). In the present study, cases of the hypoxia-ischemia group revealed a reduction in MAP2 immunostaining in hippocampal areas CA2 – CA4 and in cortical layers II-VI compared to controls. The most vulnerable regions were the hippocampal area CA4 and the cortical layers III –V. According to some studies,^[63]^ even 10 minutes of anoxia can induce a reduction of MAP2 immunoreactivity in the hippocampus.

Monoclonal antibody to neurofilament protein: it is an antibody directed against nonphosphorylated neurofilaments. It tags dendrites and the cell body of a subtype of pyramidal neurons.^[54,68]^ Leifer et al^[54]^ conducted an immunohistochemical study on autopsy cases of cerebral hypoxia/ischemia. According to the authors, the expression of SMI32 was predominantly reduced in cases of acute ischemia even when the Nissl stain revealed only slight pycnosis. The staining was not present in the areas of necrosis. However, this study had experienced limitations: a very limited number of cases studied and the presence of cases with neurodegenerative pathologies.

Albumin: Løberg et al^[69]^ conducted a study of experimental animal samples (together with several autopsy cases) in order to understand if an uptake of plasma proteins occurs in damaged neurons after ischemic/hypoxic damage. Anterior brain ischemia was induced in rats by carotid clamping and hypotension for 15 minutes, followed by recirculation for 6 hours, 24 hours, 48 hours, and 5 days. According to the authors, blood-brain barrier rupture with mild albumin extravasation was revealed 6 hours after ischemic/hypoxic damage in the lateral reticular nucleus of the thalamus, in the dorsolateral striatum and watershed area of the cerebral cortex. Previously albumin expression after ischemic/hypoxic damage has been studied by Maeda et al^[70]^ in experimental animal samples. According to the authors and their results from optical microscopy, there was no reaction to albumin for the first 12 hours after unilateral occlusion of the common carotid artery for 10 minutes as well as reperfusion. At 12 hours, the reaction was weak and limited in the CA1 subiculum region. More significant results were obtained by the same authors through the use of electron microscopy. However, it should be considered that the expression immunohistochemistry of albumin can occur as a postmortem phenomenon,^[69]^ therefore its objective application in the forensic field be extremely limited and not considered very useful. For this reason, Løberg et al^[69]^ proposed to test fibrinogen for autopsy cases of hypoxic brain damage.

Glial fibrillary acid protein (GFAP) and Vimentin: The GFAP is the main intermediate filament protein in mature astrocytes.^[71]^ Vimentin is part of the family of intermediate filament proteins and plays a key role in controlling microglia activation and neurotoxicity during cerebral ischemia.^[72]^ In the rat hippocampus after ischemia, vimentin and GFAP positive astrocytes appeared solely in the CA1 region, indicating neuronal necrosis, while GFAP positive and vimentin negative cells were observed not only in the CA1 region but in the CA3 region as well, which indicated neuronal vitality.^[1,73]^ Based on these results, vimentin could be considered a useful marker for neuronal necrosis (but not in very acute hypoxic damage). According to Loefer et al,^[54]^ a reduction in the expression of GFAP was identified in very acute lesions of a series of autopsy specimens while the lack of reactivity of this marker had increased in damage after a few days. This study, however, has considerable limitations for it was conducted on a very limited series of cases, and a part of them experienced neurodegenerative diseases as well. GFAP and vimentin have also been studied with autoptic cases.^[10,60]^ According to Kitamura,^[10]^ the proliferation of GFAP-positive and vimentin-positive cells (astrocytes and microglia) was primarily observed after hypoxic/ischemic damage with prolonged survival time. In cases that have a previous history of hypoxic damage, the author indicated a proliferation of GFAP-positive and vimentin-negative astrocytes in the CA3 and CA4 regions of the hippocampus. However, and according to this study, the immunohistochemical evaluation of GFAP cannot distinguish hypoxic/ischemic damage from postmortem alterations. In conclusion, GFAP and vimentin play a vital role if the survival time is prolonged for; they are linked to the reaction of glial cells to hypoxic/ischemic damage. Therefore, the use of these markers is considered of no use in the case of very recent hypoxic damage.

## 4 Discussion

In the field of forensic neuropathology, evidence of a hypoxic/ischemic brain injury is particularly important.^[1]^ Even if the brain is the target organ of this type of damage, at present, there are no specific neuropathological (macroscopic and histological) findings of hypoxic damage (such as drowning, hanging, carbon monoxide poisoning) or acute ischemic.^[1,4,6,74]^ According to the literature,^[1,75]^ the mechanism of death in hypoxia/ischemia is too rapid to determine the presence of vital morphological changes. Postmortem changes can alter the cerebral parenchyma rendering the assessment of brain tissue even more difficult.

In the postmortem diagnosis of ischemia/brain hypoxia, immunohistochemistry could help and overcome the limitations of conventional histology. Therefore, in-depth knowledge of cellular reactions triggered by neuronal hypoxic damage is particularly important.

Ischemia/hypoxia induces severe stress on nerve cells which leads to the activation of immediate early genes (such as c-fos) and their coding for thermal shock proteins (such as HSP70).^[76–79]^ While acute ischemic neuronal injury indicates axon sparing and selective neuronal injury (due to the release of large quantities of glutamate into the extracellular space), late neuronal death is associated with anti-apoptotic growth factors and reduced expression of microtubule-associated proteins and tubulin.^[5]^ Immunohistochemistry studies of acute hypoxic/ischemic brain damage have often been based on this knowledge.

In this perspective, the review we have proposed summarizes and considers all scientific studies relating to the immunohistochemical diagnosis of acute cerebral hypoxia/ischemia in the last 25 years or so.

In every original paper analyzed, an immunohistochemical study was performed in the brain areas most susceptible to ischemic damage, specifically in the hippocampus, cerebellum and watershed area.

By far, microtubule-associated protein 2 (MAP2) is the most researched biomarker and has provided the best results. In fact, one of the studies in the forensic field^[1]^ has revealed a reduction of MAP2 expression in cases of cerebral hypoxia (such as hanging and drowning) in which the agonic time (of survival) was a few minutes. The results of this study seem very encouraging and so the marker could be very useful in the detection of very recent cerebral hypoxia. In our opinion, further scientific confirmation studies on autopsy specimens would be needed before MAP2 is utilized in common forensic practice.

The results obtained by the study by Bartschat et al^[2]^ also seem very interesting. The authors, in fact, discovered a significant reduction in the cerebellar expression of calbindin-D28k in a Medico-Legal case of acute cerebral hypoxia (such as drowning or generic asphyxia). The survival time of the study group was only a few minutes; therefore, the marker could also be useful in supporting the diagnosis of acute hypoxia. This study can be considered an important pioneering analysis however additional confirmatory studies would be required.

With regards to a large part of the other markers analyzed (such as HIF-1 alpha, HSP70, vimentin, VEGF and GFAP) the results were very poor and their use in the detection of acute cerebral hypoxia/ischemia is not possible.

A problem that emerged from the original papers studied was that the markers were often tested on a small number of autopsy cases and further confirmation studies were carried out in a limited number. As a result, the studied markers have attracted just a theoretical interest (in some cases, furthering the pathological mechanism triggered by hypoxic/ischemic brain damage) but they do not have a significant application in forensic practice.

In studies on animal samples, the experimental model was inconclusive and could not be compared to any specific autopsy case. For example, during the preparation of tissue samples, the occlusion times of cerebral arteries were long (approximately 1 hour), while in human beings the damage from hypoxic phenomena related to asphyxia (in drowning, for example) continues for only several minutes. Therefore, the sample obtained through animal studies could have a different or enhanced immunohistochemical expression compared to autopsy cases. Therefore, in the absence of other confirmatory studies on autopsy specimens, these experimental studies performed on animals (although they have particular theoretical and even clinical interests) cannot serve a forensic purpose.

Another particular problem in the forensic field is postmortem alteration due to autolysis and putrefaction. In this regard, the quality of immunohistochemical staining always depends on the duration of autolysis, namely, on the period of the moment of death of an individual and the fixation of the samples.^[80,81]^ Autolysis and putrefaction cause tissue alteration with the degeneration of protein structures. These processes depend on various factors of both the corpse (type of death, physical constitution) and environmental factors (temperature, humidity, ventilation).^[28,47,82]^

It is not always possible to perform an autopsy immediately after death for the discovery of a corpse can occur sometime after death and/or due to the necessity of authorization from judicial authorities to perform the autopsy. For example, due to the movements of a corpse in water, its discovery could occur within a few days of death by drowning, therefore, severely limiting an immunohistochemical investigation. However, scientific studies in the literature often analyze cases with a very limited postmortem interval, and the immunohistochemical expression of markers in autolytic or initially putrefied samples is not evaluated. Therefore, the attempt to fully understand the resistance of the markers to post-lethal alterations is difficult when it comes to positive scientific significance.

To sum up, despite many promising studies, at present, it is difficult to identify reliable and confirmed biomarkers from multiple studies in order to support a postmortem diagnosis of acute cerebral hypoxia/ischemia. Without a doubt, MAP2 is the most researched marker in the literature and the results obtained have proven to be quite useful however, only a few select studies have used human brain samples.^[1]^

Moreover, there are significant limitations in the studies analyzed, mainly in relation to the limited number of autopsy cases studied and the alterations due to autolytic and putrefactive phenomena.

However, evidence of cerebral hypoxia is often important in judicial autopsies, especially in cases of violent mechanical asphyxia. The results of this review could also be useful for judicial court.

In conclusion, for the time being, proof of cerebral hypoxia is particularly difficult if the survival time is very short which usually occurs in asphyxia. In these cases, it is necessary to accurately follow the indications suggested in the literature,^[5,6,16–20]^ and always consider every brain area that is most susceptible to hypoxic damage.

Only a few select immunohistochemistry studies have been performed to provide support in the detection of acute cerebral ischemia/hypoxia. The results from several of the biomarkers seem promising however further confirmatory studies are strongly recommended when it comes to their application in common forensic practice.

## Author contributions

**Conceptualization:** Rosario Barranco, Francesco Ventura.

**Data curation:** Rosario Barranco, Alessandro Bonsignore.

**Formal analysis:** Alessandro Bonsignore.

**Methodology:** Rosario Barranco.

**Supervision:** Francesco Ventura.

**Validation:** Francesco Ventura.

## References

[1] Kuhn J Meissner C Oehmichen M. Microtubule-associated protein 2 (MAP2) – a promising approach to diagnosis of forensic types of hypoxia–ischemia. Acta Neuropathol 2005;110:579–86. 16328528 10.1007/s00401-005-1090-9

[2] Bartschat S Fieguth A Könemann J Schmidt A Bode-Jänisch S. Indicators for acute hypoxia--an immunohistochemical investigation in cerebellar Purkinje-cells. Forensic Sci Int 2012;223:165–70. 22980140 10.1016/j.forsciint.2012.08.023

[3] Oehmichen M Meissner C von Wurmb-Schwark N Schwark T. Methodical approach to brain hypoxia/ischemia as a fundamental problem in forensic neuropathology. Leg Med (Tokyo) 2003;5:190–201. 14602162 10.1016/s1344-6223(03)00077-4

[4] Oehmichen M. Brinkmann B Puschel K. Neuropathologie der forensisch relevanten Formen des Erstickens. Ersticken, Fortschritte in der Beweisfuhrung 1st ed Berlin: Springer; 1990. 151–7.

[5] Oechmichen M Meissner C. Cerebral hypoxia and ischemia: the forensic point of view: a review. J Forensic Sci 2006;51:880–7. 16882233 10.1111/j.1556-4029.2006.00174.x

[6] Springer Verlag, Oehmichen M Auer RN König HG. Forensic Neuropathology and Associated Neurology Berlin. 2005.

[7] Auer RN. Oehmichen M. Pure hypoxic and ischemic brain insults. Brain hypoxia and ischemia. Research in legal medicine, Vol. 24. Lubeck: Schmidt-Romhild; 2000. 27–39.

[8] Rahaman P Del Bigio MR. Histology of brain trauma and hypoxia-ischemia. Acad Forensic Pathol 2018;8:539–54. 31240058 10.1177/1925362118797728 PMC6490582

[9] Schmidt-Kastner R. Genomic approach to selective vulnerability of the hippocampus in brain ischemia-hypoxia. Neuroscience 2015;309:259–79. 26383255 10.1016/j.neuroscience.2015.08.034

[10] Kitamura O. Immunohistochemical investigation of hypoxic/ischemic brain damage in forensic autopsy cases. Int J Legal Med 1994;107:69–76. 7819122 10.1007/BF01225492

[11] Graham DI Adams JH Duchen LW. Love S Perry A Ironside J Budka H. Hypoxia and vascular disorders. Greenfield's neuropathology. London: Edward Arnold; 1992. 153–268.

[12] Horn M Schlote W. Delayed neuronal death and delayed neuronal recovery in the brain following global ischemia. Acta Neuropathol 1992;85:79–87. 1285498 10.1007/BF00304636

[13] Kirino T Sano K. Selective vulnerability in the gerbil hippocampus following transient ischemia. Acta Neuropathol 1984;62:201–8. 6695554 10.1007/BF00691853

[14] Kirino T Sano K. Fine structural nature of delayed neuronal death following ischemia in the gerbil hippocampus. Acta Neuropathol 1984;62:209–18. 6695555 10.1007/BF00691854

[15] Petito CK Feldmann E Pulsinelli WA Plum F. Delayed hippocampal damage in humans following cardiorespiratory arrest. Neurology 1987;37:1281–6. 3614648 10.1212/wnl.37.8.1281

[16] Esiri M Perl D. Oppenheimer's Diagnostic Neuropathology: A Practical Manual. Third ed London: Hodder Arnold; 2006.

[17] Whitwell HL. Forensic Neuropathology. London: Hodder Arnold; 2005.

[18] Leestma JE. Forensic Neuropathology. Third edition Boca Raton: CRC Press; 2014.

[19] Ellison D Love S Cardao Chimelli LM. Neuropathology. A reference text of CNS pathology. Third ed. Edinburgh: Mosby Elsevier; 2013.

[20] Itabashi HH Andrews JM Tomiyasu U. Forensic Neuropathology A Practical Review of the Fundamentals. Amsterdam: Elsevier; 2007.

[21] Salama M Mohamed WM. Tau protein as a biomarker for asphyxia: a possible forensic tool? Appl Transl Genom 2016;9:20–2. 27354936 10.1016/j.atg.2016.03.001 PMC4912031

[22] Kambe T Motoi Y Inouem R. Differential regional distribution of phosphorylated tau and synapse loss in the nucleus accumbens in tauopathy model mice. Neurobiol Dis 2011;42:404–14. 21324362 10.1016/j.nbd.2011.02.002

[23] Villamil-Ortiz JG Cardona-gomez GP. Comparative analysis of autophagy and tauopathy related markers in cerebral ischemia and Alzheimer's disease animal models. Front Aging Neurosci 2015;7:01–14. 10.3389/fnagi.2015.00084 PMC4436888 26042033

[24] Li DR Zhu BL Ishikawa T. Immunohistochemical distribution of S-100 protein in the cerebral cortex with regard to the cause of death in forensic autopsy. Leg Med (Tokyo) 2006;8:78–85. 16338157 10.1016/j.legalmed.2005.09.002

[25] Isobe T Takahasi K Okuyama T. S100a protein is present in neurons of the central and peripheral nervous system. J Neurochem 1984;43:1494–6. 6387052 10.1111/j.1471-4159.1984.tb05415.x

[26] Donato R. S100: a multigenic family of calcium-modulated proteins of the EF-hand type with intracellular and extracellular functional roles. Int J Biochem Cell Biol 2001;33:637–68. 11390274 10.1016/s1357-2725(01)00046-2

[27] Kligman D Hilt DC. The S100 protein family. Trends Biochem Sci 1988;13:437–43. 3075365 10.1016/0968-0004(88)90218-6

[28] Barranco R Ventura F. Immunohistochemistry in the detection of early myocardial infarction: systematic review and analysis of limitations because of autolysis and putrefaction. Appl Immunohistochem Mol Morphol 2018;28:95–102. 10.1097/PAI.0000000000000688 32044877

[29] Haimoto H Hosoda S Kato K. Differential distribution of immunoreactive S100a and S100b proteins in normal nonnervous human tissues. Leb Invest 1987;57:489–98. 3316838

[30] Haimoto H Kato K. S100a a calcium-binding protein, is localized in the slow-twitch muscle fiber. J Neurochem 1987;48:917–23. 3543224 10.1111/j.1471-4159.1987.tb05604.x

[31] Kato K Kimura S. S100a protein is mainly located in the heart and striated muscles. Biochem Biophys Acta 1985;17:146–50. 10.1016/0304-4165(85)90196-5 4052452

[32] Dimopoulou I Korfias S Dafni U. Protein S-100b serum levels in trauma-induced brain death. Neurology 2003;60:947–51. 12654958 10.1212/01.wnl.0000049931.77887.7f

[33] Raabe A Grolms C Sorge O Zimmermann M Seifert V. Serum S-100B protein in severe head injury. Neurosurgery 1999;45:477–83. 10493369 10.1097/00006123-199909000-00012

[34] Regner A Kaufman M Friedman G. Increased serum S100b protein concentrations following severe head injury in human: a biochemical marker of brain death? Neuroreport 2001;26:691–4. 10.1097/00001756-200103260-00015 11277565

[35] Ishida K Gohara T Kawata R. Are serum S100b proteins and neuron-specific enolase predictors of cerebral damage in cardiovascular surgery? J Cardiothorac Vasc Anesth 2003;17:04–9. 10.1053/jcan.2003.2 12635053

[36] Shaaban-Ali M Harmer M Vaughn RS. Serum S100 protein as a marker of cerebral damage during cardiac surgery. Br J Anaesth 2000;85:287–98. 10992840 10.1093/bja/85.2.287

[37] Woertgen C Rothoerl RD Holzschuh M. Comparison of serial S-100 and NSE serum measurements after severe head injury. Acta Neurochir 1997;139:1161–5. 9479423 10.1007/BF01410977

[38] Ingebrigtsen T Romner B Marup-Jensen S. The clinical value of serum S-100 protein measurements in minor head injury: a Scandinavian Multicentre Study. Brain Inj 2000;14:1047–55. 11147577 10.1080/02699050050203540

[39] Ingebrigtsen T Romner B Kongstad P Langbakk B. Increased serum concentrations of protein S-100 after minor head injury: a biochemical serum marker with prognostic value? J Neurol Neurosurg Psychiatry 1995;59:103–4. 10.1136/jnnp.59.1.103-a PMC1073618 7608699

[40] Romner B Ingebrigtsen T Kongstad P Børgesen SE. Traumatic brain damage: serum S-100 protein measurements related to neuroradiological findings. J Neurotrauma 2000;17:641–7. 10972241 10.1089/089771500415391

[41] Katsetos CD Spandou E Legido A. Acute hypoxia-induced alterations of calbindin-D28k immunoreactivity in cerebellar Purkinje cells of the guinea pig fetus at term. J Neuropathol Exp Neurol 2001;60:470–82. 11379822 10.1093/jnen/60.5.470

[42] Blanco Pampín J García Rivero SA Otero Cepeda XL. Immunohistochemical expression of HIF-1alpha in response to early myocardial ischemia. J Forensic Sci 2006;51:120–4. 16423235 10.1111/j.1556-4029.2005.00014.x

[43] Whitelaw ML Gustafsson JA Poellinger L. Identification of transactivation and repression functions of the dioxin receptor and its basic helix-loophelix/PAS partner factor Arnt: inducible versus constitutive modes of regulation. Mol Cell Biol 1994;14:8343–55. 7969169 10.1128/mcb.14.12.8343-8355.1994 PMC359373

[44] Wang GL Jiang BH Rue EA. Hypoxia-inducible factor 1 is a basic-helix-PAS heterodimer regulated by cellular O2 tension. Proc Nat Acad Sci 1995;92:5510–4. 7539918 10.1073/pnas.92.12.5510 PMC41725

[45] Ferrara N. Molecular and biological properties of vascular endothelial growth factor. J Mol Med 1999;77:527–43. 10494799 10.1007/s001099900019

[46] Ferrara N. Vascular endothelial growth factor and the regulation of angiogenesis. Recent Prog Horm Res 2000;55:15–36. 11036931

[47] Barranco R Ventura F. Immunohistochemistry in the postmortem diagnosis of sepsis: a systematic review. Appl Immunohistochem Mol Morphol 2020;28:571–8. 31290786 10.1097/PAI.0000000000000790

[48] Fan X Heijnen CJ van der Kooij MA Groenendaal F van Bel F. The role and regulation of hypoxia-inducible factor-1alpha expression in brain development and neonatal hypoxic–ischemic brain injury. Brain Res Rev 2009;62:99–108. 19786048 10.1016/j.brainresrev.2009.09.006

[49] Lopez-Barneo J Pardal R Ortega-Saenz P. Cellular mechanism of oxygen sensing. Annu Rev Physiol 2001;63:259–87. 11181957 10.1146/annurev.physiol.63.1.259

[50] Sanz O Estrada A Ferrer I Planas AM. Differential cellular distribution and dynamics of HSP70, cyclooxygenase-2, and c-Fos in the rat brain after transient focal ischemia or kainic acid. Neuroscience 1997;80:221–32. 9252233 10.1016/s0306-4522(97)00089-4

[51] Feng L Sun W Xia Y. Cloning two isoforms of rat cyclooxygenase: diVerential regulation of their expression. Archs Biochem Biophys 1993;307:361–8. 10.1006/abbi.1993.1601 8274023

[52] Planas AM Soriano MA Rodriguez-Farre’ E Ferrer I. Induction of cyclooxygenase-2 mRNA and protein following transient focal ischemia in the rat brain. Neurosci Lett 1995;200:187–90. 9064608 10.1016/0304-3940(95)12108-g

[53] Herrera DG Robertson HA. Activation of c-fos in the brain. Prog Neurobiol 1996;50:83–107. 8971979 10.1016/s0301-0082(96)00021-4

[54] Leifer D Kowall NW. Immunohistochemical patterns of selective cellular vulnerability in human cerebral ischemia. J Neurol Sci 1993;119:217–28. 8277338 10.1016/0022-510x(93)90137-n

[55] Barranco R Castiglioni C Ventura F. Immunohistochemical expression of P-selectin, SP-A, HSP70, aquaporin 5, and fibronectin in saltwater drowning and freshwater drowning. Int J Legal Med 2019;133:1461–7. 31222534 10.1007/s00414-019-02105-1

[56] Han SG Castranova V Vallyathan V. Heat shock protein 70 as an indicator of early lung injury caused by exposure to arsenic. Mol Cell Biochem 2005;277:153–64. 16132727 10.1007/s11010-005-5874-y

[57] Georgopoulos C Welch WJ. Role of the major heat shock proteins as molecular chaperons. Annu Rev Cell Biol 1993;9:601–34. 8280473 10.1146/annurev.cb.09.110193.003125

[58] Ciocca DR Oesterreich S Chamness GC. Biological and clinical implication of heat shock protein 27000 (Hsp27): a review. J Nat Cancer Inst 1993;85:1558–70. 8411230 10.1093/jnci/85.19.1558

[59] Murphy ME. The HSP70 family and cancer. Carcinogenesis 2013;34:1181–8. 23563090 10.1093/carcin/bgt111 PMC3670260

[60] Kubo S Kitamura O Orihara Y Ogata M Tokunaga I Nakasono I. Immunohistochemical diagnosis and significance of forensic neuropathological changes. J Med Invest 1998;44:109–19. 9597798

[61] Hirokawa N. Microtubule organization and dynamics dependent on microtubule-associated proteins. Curr Opin Cell Biol 1994;6:74–81. 8167029 10.1016/0955-0674(94)90119-8

[62] Kitagawa K Matsumoto M Niinobe M. Microtubule-associated protein 2 as a sensitive marker for cerebral ischemic damage-immunohistochemical investigation of dendritic damage. Neuroscience 1989;31:401–11. 2797444 10.1016/0306-4522(89)90383-7

[63] Kwei S Jiang C Haddad GG. Acute anoxia-induced alterations in MAP2 immunoreactivity and neuronal morphology in rat hippocampus. Brain Res 1993;620:203–10. 8369956 10.1016/0006-8993(93)90157-i

[64] Malinak C Silverstein FS. Hypoxic-ischemic injury acutely disrupts microtubule-associated protein 2 immunostaining in neonatal rat brain. Biol Neonate 1996;69:257–67. 8724654 10.1159/000244319

[65] Ota A Ikeda T Ikenoue T Toshimori K. Sequence of neuronal responses assessed by immunohistochemistry in the newborn rat brain after hypoxia-ischemia. Am J Obstet Gynecol 1997;177:519–26. 9322617 10.1016/s0002-9378(97)70139-x

[66] Yanagihara T Brengman JM Mushynski WE. Differential vulnerability of microtubule components in cerebral ischemia. Acta Neuropathol 1990;80:499–505. 2251907 10.1007/BF00294610

[67] Akulinin VA Dahlstrom A. Quantitative analysis of MAP2 immunoreactivity in human neocortex of three patients surviving after brain ischemia. Neurochem Res 2003;28:373–8. 12608711 10.1023/a:1022401922669

[68] Campbell MJ Morrison JH. Monoclonal antibody to neurofilament protein (SMI-32) labels a subpopulation of pyramidal neurons in the human and monkey neocortex. J Comp Neurol 1989;282:191–205. 2496154 10.1002/cne.902820204

[69] Løberg EM Karlsson BR Torvik A. Neuronal uptake of plasma proteins after transient cerebral ischemia/hypoxia. Immunohistochemical studies on experimental animals and human brains. APMIS 1993;101:777–83. 8267954 10.1111/j.1699-0463.1993.tb00179.x

[70] Maeda M Akai F Nishida S Yanagihara T. Intracerebral distribution of albumin after transient cerebral ischemia: light and electron microscopic immunocytochemical investigation. Acta Neuropathol 1992;84:59–66. 1502882 10.1007/BF00427216

[71] Middeldorp J Hol EM. GFAP in health and disease. Prog Neurobiol 2011;93:421–43. 21219963 10.1016/j.pneurobio.2011.01.005

[72] Jiang SX Slinn J Aylsworth A. Hou ST J Neurochem. Vimentin participates in microglia activation and neurotoxicity in cerebral ischemia 2012;122:764–74. 10.1111/j.1471-4159.2012.07823.x 22681613

[73] Morioka T Kalehua AN Streit WJ. The microglial reaction in the rat dorsal hippocampus following transient forebrain ischemia. J Cereb Blood Flow Metab 1991;11:966–73. 1719009 10.1038/jcbfm.1991.162

[74] Schmidt-Romhild, Lubeck, Oehmichen M Meissner C. Oehmichen M. Forensic neuropathological aspects of cerebral anoxia/ischemia and hypoxia/hypoxemia. Brain hypoxia and ischemia. Research in legal medicine, 1st edn, vol 24 2000. 13–25.

[75] Knight B Forensic pathology. 3rd edn London: Arnold; 2004.

[76] Vass K Welch WJ Nowak TS. Localization of 70-kDa stress protein induction in gerbil brain after ischemia. Acta Neuropathol (Berlin) 1988;77:128–35. 10.1007/BF00687422 3227811

[77] Chopp M Li Y Dereski MO Levine SR Yoshida Y Garcia JH. Neuronal injury and expression of 72-kDa heat-shock protein after forebrain ischemia in the rat. Acta Neuropathol (Berlin) 1991;83:66–71. 10.1007/BF00294432 1792866

[78] Kalimo H Kaste M Haltia M. Graham DI Lantos PL. Vascular diseases. Greenfield's neuropathology. London: Arnold; 2002. 281–355.

[79] Nowak TS Jr Osborne OC Suga S. Stress protein and proto-oncogene expression as indicators of neuronal pathophysiology after ischemia. Progr. Brain Res 1993;96:195–208. 10.1016/s0079-6123(08)63267-7 8332741

[80] Dobaczewski M Gonzalez-Quesada C Frangogiannis NG. The extracellular matrix as a modulator of the inflammatory and reparative response following myocardial infarction. J Mol Cell Cardiol 2010;48:504–11. 19631653 10.1016/j.yjmcc.2009.07.015 PMC2824059

[81] Kyclova J Rotterova P Dvorak K. Effect of fixation and autolysis on immunohistochemical detection of CD antigens. Scripta Medica 2004;77:63–74.

[82] Pelstring RJ Allred DC Esther RJ. Differential antigen preservation during autolysis. Hum Pathol 1991;22:237–41. 1706304 10.1016/0046-8177(91)90156-j

